# Niobium-Doped Hydroxyapatite Bioceramics: Synthesis, Characterization and *In Vitro* Cytocompatibility

**DOI:** 10.3390/ma8074191

**Published:** 2015-07-09

**Authors:** Nádia S. V. Capanema, Alexandra A. P. Mansur, Sandhra M. Carvalho, Alexandra R. P. Silva, Virginia S. Ciminelli, Herman S. Mansur

**Affiliations:** Center of Nanoscience, Nanotechnology and Innovation-CeNano^2^I, Department of Metallurgical and Materials Engineering, Federal University of Minas Gerais/UFMG, Av. Antônio Carlos, 6627 Escola de Engenharia, Belo Horizonte/MG 31.270-901, Brazil; E-Mails: nsvnadia@gmail.com (N.S.V.C.); aapiscitelli@uol.com.br (A.A.P.M.); sandhra.carvalho@gmail.com (S.M.C.); alebiomat@gmail.com (A.R.P.S.); ciminelli@demet.ufmg.br (V.S.C.)

**Keywords:** biomaterial, bioceramic, bioactive ceramic, calcium phosphate, doped hydroxyapatite, bone tissue engineering

## Abstract

Doping calcium phosphates with ionic species can play an important role in biological responses promoting alkaline phosphatase activity, and, therefore inducing the generation of new bone. Thus, in this study, the synthesis of niobium-doped hydroxyapatite (Nb-HA) nanosize particles obtained by the precipitation process in aqueous media followed by thermal treatment is presented. The bioceramics were extensively characterized by X-ray diffraction, wavelength dispersive X-ray fluorescence spectrometry, Fourier transform infrared spectroscopy, scanning electron microscopy/energy dispersive X-ray spectroscopy analysis, transmission electron microscopy, atomic force microscopy and thermal analysis regarding their chemical composition, structure and morphology. The results showed that the precipitate dried at 110 °C was composed of amorphous calcium phosphate and HA, with polidisperse particles ranging from micro to nano dimensions. After the thermal treatment at 900 °C, the bioceramic system evolved predominantly to HA crystalline phase, with evident features of particle sintering and reduction of surface area. Moreover, the addition of 10 mol% of niobium salt precursor during the synthesis indicated the complete incorporation of the Nb(V) species in the HA crystals with detectable changes in the original lattice parameters. Furthermore, the incorporation of Nb ions caused a significant refinement on the average particle size of HA. Finally, the preliminary cytocompatibility response of the biomaterials was accessed by human osteoblast cell culture using MTT and resazurin assays, which demonstrated no cytotoxicity of the Nb-alloyed hydroxyapatite. Thus, these findings seem promising for developing innovative Nb-doped calcium phosphates as artificial biomaterials for potential use in bone replacements and repair.

## 1. Introduction

Bones, an important part of our skeletal system, play a key role in our lives, supporting our bodies and enabling us to have mobility. Since the early days of Hippocrates (500 B.C.) in ancient Greece, it has been recognized that bone is a very dynamic tissue with a unique capacity to heal and remodel under appropriate conditions without leaving a wound. This set of properties associated with its capacity to withstand load bearing makes bone a very complex system to be substituted using synthetic materials. Thus, it is a great challenge to mimick all of the bone functions when it is damaged or injured by accidents and diseases [1,2,3,4,5]. The regeneration of bone tissue using the body’s own self-healing mechanisms is an ideal approach for bone repair, which is the major goal of tissue engineering, restoring diseased or impaired tissue to its original state and function, reducing the need for transplants and replacements. However, when an area of damaged bone is excessively large for self-repair healing, the injured site must be repaired using alternative materials, such as autografts, allografts and artificial materials. Additionally, an increasing clinical demand for synthetic and artificial bone substitutes has been observed due to the rapidly aging population worldwide [1,6,7]. Currently, in order to address this problem, there are several classes of synthetic bone grafting biomaterials for *in vivo* applications, such as natural coral-derived materials, bovine demineralized bone, human demineralized bone matrix, bioactive glasses, glass-ceramics, alumina-based ceramic [8], hybrids [2,3,4,9] and calcium orthophosphates (CaP) [10,11,12,13,14,15]. All of these biomaterials need to be biocompatible and osteoconductive for cell proliferation and guiding bone tissue growth leading to tissue repair and remodeling. For that reason, over the last four decades, bioactive ceramic materials have gained highest attention from the scientific community and professionals because of their extraordinary potential use as suitable bone substitutes. Commonly, bioceramics are considered ceramics that are designed to induce specific biological activity for repairing damaged organs. Since the discovery of Bioglass^®^ [16], many researchers have developed numerous types of bioactive ceramics, such as hydroxyapatite (Ca_10_(PO_4_)_6_(OH)_2_) [17,18] and glass-ceramic [19]. Despite the fact that materials science technology has resulted in unquestionable advances in the field of bone replacement medicine, no totally satisfactory bone substitute, which fulfills all requirements, has been developed yet. Hence, the development of bioactive materials that show not only bioactivity but also mechanical properties similar to living bone is still much needed [1]. Among several alternatives of ceramic-based materials for bone replacement and repair, bioceramics made of calcium phosphates (CaPs) appear very promising due to both excellent biocompatibility and their ability to bond to living bone in the body, which is intrinsically related to their abundance in nature and presence in mammalian calcified tissues [6,7]. Hydroxyapatite (HA) is the most well-known CaP material, since it is crystallographically and chemically similar to the mineral phase of human bone. Therefore, it has been intensively studied for use as biomaterial and scaffold for bone tissue regeneration. However, it is important to note that native bone apatite differs from stoichiometric HA in a number of ways, including non-stoichiometry, nanosized crystal dimensions, and a relative crystallinity when assuming 100% for stoichiometric HA. The non-stoichiometry of biological apatites is mostly caused from the incorporation of foreign ions into the crystal lattice [20]. Interestingly, studies confirmed that substituting ions (anions or cations) present in native hard tissues such as strontium (Sr), magnesium (Mg), zinc (Zn), [20] and niobium (Nb) [21,22,23] into CaPs can lead to beneficial effects on biomaterial properties, such as the degree of structural order (*i.e.*, crystallinity), morphology, thermal stability, solubility, mechanical properties, degradability, surface charge, and dissolution rate under physiological conditions. Furthermore, the doping with ionic species can play an important role in the biological responses of bone cells [20]. Masato Tamai *et al.* [21] reported that Nb(V) incorporated as niobates to biphasic calcium phosphate (HA and β-tricalcium phosphate, β-TCP) significantly promoted the calcification of normal human osteoblasts and has the potential to promote alkaline phosphatase (ALP) activity, an important factor in the generation of new bone. Consequently, Nb(V) species can be considered as key dopants for the incorporation in HA, as most niobium salt precursors (e.g., NbCl_5_) undergo hydrolysis in alkaline aqueous medium leading to the formation of oxyanions (generic formula Nb_x_O_y_^z−^) instead of Nb^5+^ [21,23,24].

Thus, the main goal of this study was the synthesis and characterization of niobium-modified bioceramics for potential use as biomaterial in bone tissue repair. Although there are few published studies in this field, no research was found in the consulted literature where a systematic and an extensive characterization of morphology, structure, and the cytotoxicity of Nb-doped HA produced by co-precipitation method under the same experimental conditions have been performed.

## 2. Results and Discussion

The results of the synthesized bioceramics are presented in the next sections and the samples were identified as follows: HA_110 (hydroxyapatite dried at 110 °C); Nb-HA_110 (Nb-doped hydroxyapatite dried at 110 °C); HA_900 (hydroxyapatite sintered at 900 °C); and Nb-HA_900 (Nb-doped hydroxyapatite sintered at 900 °C).

### 2.1. Energy Dispersive X-ray Spectroscopy Analysis (EDX) and Wavelength Dispersive X-ray Fluorescence Spectrometry (WD-XRF)

**Figure 1:**
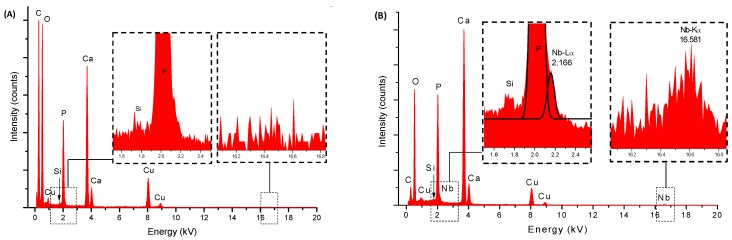
EDX spectra of HA_900 (**A**) and Nb-HA_900 (**B**).

**Figure 2:**
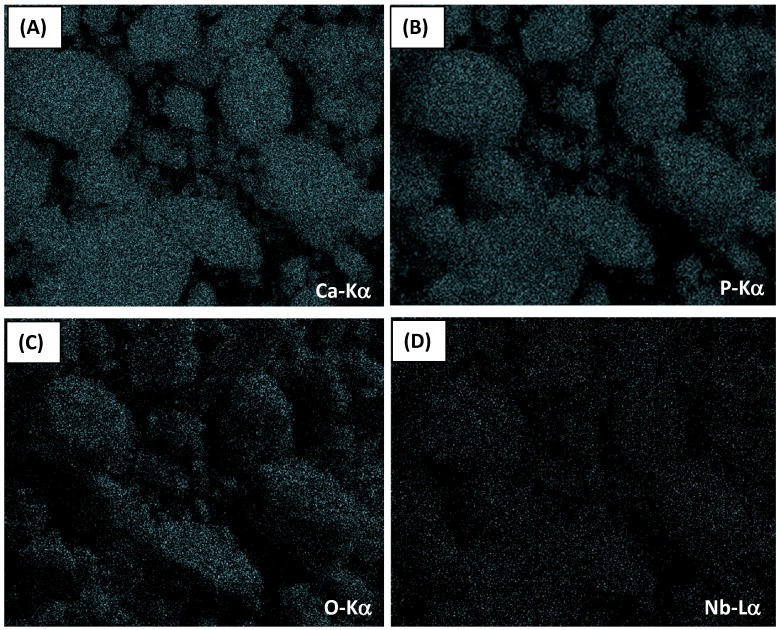
EDX image mapping of Ca (Kα) (**A**); P (Kα) (**B**); O (Kα) (**C**); and Nb (Lα) (**D**) in Nb-HA_900 sample (magnification, 2400×).

The chemical analyses evaluated by EDX of HA_900 and Nb-HA_900 powder samples are shown in Figure 1A. As expected, the major element components found in the analysis were phosphorus (P), calcium (Ca), and oxygen (O) that formed the calcium phosphate besides copper (Cu) and carbon (C) peaks components of the TEM grids, and Si from the detector. The Ca/P atomic ratios from EDX analysis were calculated and compared to the hydroxyapatite stoichiometric molar ratio of 1.67. The results ranged from 1.5 to 2.0 (HA_110: Ca/P = 1.75 ± 0.04 and HA_900: Ca/P = 1.75 ± 0.06), in good agreement with Ca/P ratio of pure hydroxyapatite reported in the literature [25]. The EDX spectrum of Nb-HA_900 (Figure 1B) showed the presence of niobium peaks, confirming the presence of this doping element in the material produced. In addition, the EDX image mapping of Ca (Figure 2A), P (Figure 2B), O (Figure 2C), and Nb (Figure 2D) showed that these elements are uniformly distributed in the sample, with no detectable segregation or any other phase present, as previously reported by Tamai *et al.* [21].

**Figure 3:**
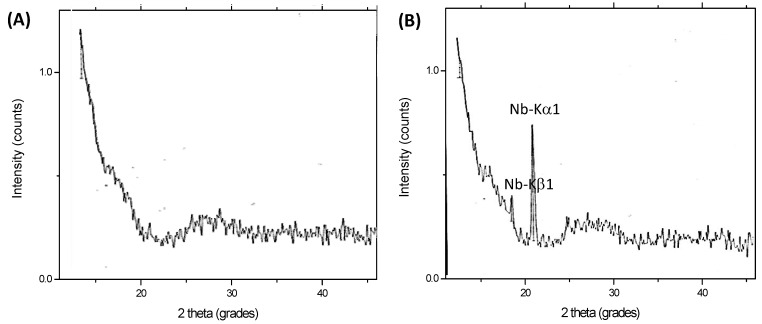
WD-XRF spectra of HA_900 (**A**) and Nb-HA_900 (**B**).

Figure 3 shows the WD-XRF spectra of HA_900 (A) and Nb-HA_900 (B) samples. The quantitative chemical analysis of Nb by WD-XRF and calibration curves revealed a concentration of 9.0 ± 0.5 mol%, in good agreement with theoretical value of 10 mol% used for sample preparation. Therefore, these results have given strong evidence that niobium was successfully incorporated into the HA bioceramic matrices.

### 2.2. X-ray Diffraction (XRD) Analysis

**Figure 4:**
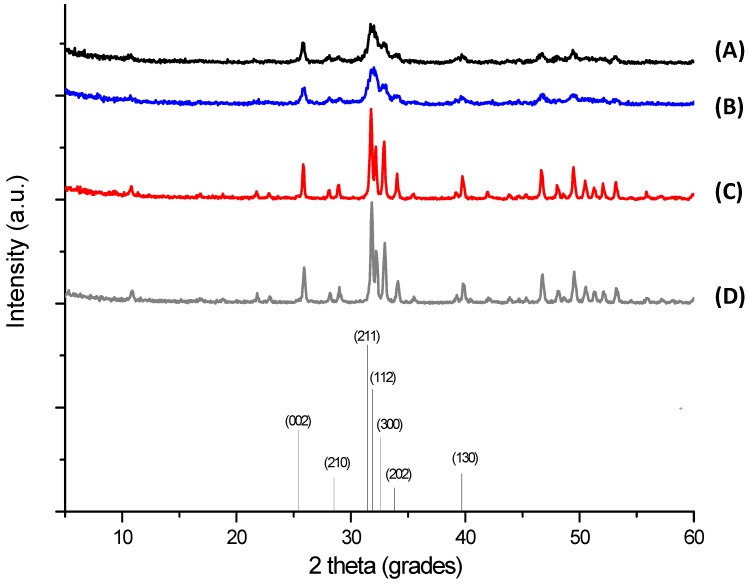
XRD patterns of HA_110 (**A**); Nb-HA_110 (**B**); HA_900 (**C**); and Nb-HA_900 (**D**). Bottom: reference of main peaks of HA (JCPDS, ICDD—96-900-3549).

The XRD patterns of the synthesized precipitates are shown in Figure 4. They revealed that the powders were HA, in agreement with those published in the literature and with the characteristic peaks consistent with International Centre for Diffraction Data (JCDS 2001) file for HA (ICDD—96-900-3549). The presence of secondary phases, such as α-TCP, β-TCP, CaO, and others, were not detected. The results of the samples dried at 110 °C (Figure 4A,B) showed the presence of a poorly crystalline carbonated HA, which is usual for HA powders prepared via the wet chemical route [6]. The crystallinity was increased by the thermal treatment performed at 900 °C (Figure 4C,D), observed by the sharper and narrower diffraction peaks, which indicated a structural reorganization in the material upon heating.

Table 1 summarizes the results of crystallite size, lattice parameters, and unit cell volume. The size of HA crystallites were in the nanoscale range estimated using the Scherrer equation for the (211). In addition, as expected, HA_900 and Nb-HA_900 powders presented larger crystallite sizes due to the heat treatment at 900 °C, which caused crystal growth [26]. The lattice parameters of the sintered crystalline samples of HA and Nb-HA were calculated indicating the increase in the sizes of “a” and “c” parameters caused by the modification of the bioceramic with Nb leading to an overall “expansion” of the unit cell volume compared to the undoped HA. As Nb(V) species are present in aqueous medium predominantly as negatively charged niobates [21], with ionic radius higher than PO_4_^3−^ [21], the increase in lattice parameters of Nb-dopped HA suggests the effective substitution of PO_4_^3−^ sites by niobates into the hydroxyapatite structure.

**Table 1:** Lattice parameters of synthesized powders.

Sample	Crystallite size [Å] *	Lattice parameters [Å] *		Unit cell volume [Å^3^]
-	-	a = b	c	-
HA_110	94.7 ± 4.1	-	-	-
Nb-HA_110	86.9 ± 3.4	-	-	-
HA_900	365.3 ± 6.3	9.419 ± 0.004	6.874 ± 0.003	528.1
Nb-HA_900	365.3 ± 6.5	9.432 ± 0.006	6.892 ± 0.000	531.0

Note: * mean ± standard deviation.

### 2.3. Thermal Analysis

**Figure 5:**
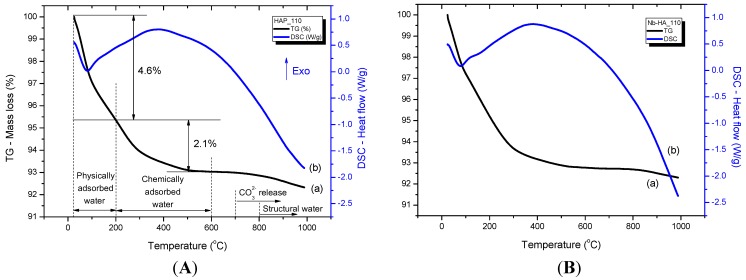
TG (a) and DSC (b) curves of HA_110 (**A**) and Nb-HA_110 (**B**).

**Figure 6:**
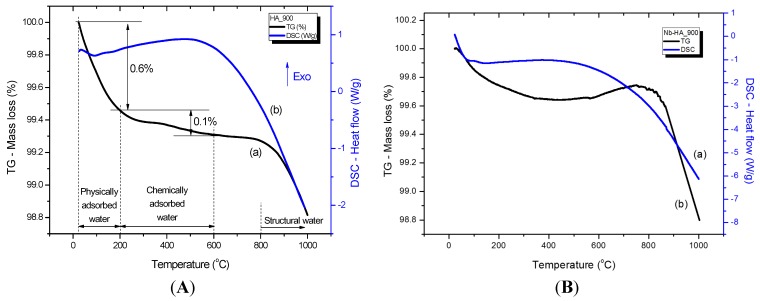
TG (a) and DSC (b) curves of HA_900 (**A**) and Nb-HA_900 (**B**).

Figure 5 and Figure 6 illustrate the thermal analyses (Thermogravimetric, TG, curve (a) and Differential Scanning Calorimetry, DSC, curve (b)) of HA_110 (A) and Nb-HA_110 (B) and HA_900 (A) and Nb-HA_900 (B) bioceramics, respectively. Three main steps can be observed in the TG curve of HA_110 (Figure 5Aa). The first region of decrease in mass ranges from 20 to 200 °C with a mass loss of approximately 4.6%, which was due to the evaporation of water physically adsorbed on the surface of HA [27,28]. This event may be associated with the endothermic peak centered at approximately 80 °C in the DSC curve (Figure 5Ab). The second region starts at the temperature of approximately 200 °C and ends at 600 °C with a corresponding mass loss of approximately 2.1%, which is attributed to the removal of chemically adsorbed water [27,28] that is irreversibly. In this range of temperature (200 to 600 °C) endothermic and exothermic events take place. However, the exothermic broad band dominates the DSC curve, which corresponds to a structural rearrangement on crystal lattice as detected by XRD. By maintaining the heating process, the CO_3_^2−^ that occupies the hydroxide or phosphate sites can be released at the temperature of approximately 700 °C without considerable mass loss. At temperatures above 800 °C, the gradual dehydration hydroxyapatite with the release of hydroxyls results in a reduction of mass (~0.5%) of the bioceramic powder sample [27,28]. The thermal profiles of the Nb-doped bioceramic samples that were dried at 110 °C (Figure 5B) did not present considerable changes in comparison with the HA_110 samples (undoped, Figure 5A).

For HA_900 (Figure 6A), only a small amount of physically (0.6%) and chemically (0.1%) adsorbed water was released, as a consequence of the thermal treatment at 900 °C, as an indicative of the relative thermal stability of the sintered bioceramic. Minor mass loss due to dehydration of HA at temperatures higher than 800 °C was also detected (~0.2%). A similar behavior was observed for Nb-HA_900 (Figure 6B), but some slight fluctuation in mass gain and mass loss at approximately 800 °C can be noted. That is mostly associated with chemical reactions occurring simultaneously forming unstable species as reported in the literature [29,30,31].

### 2.4. Fourier Transform Infrared Spectroscopy (FTIR)

**Figure 7:**
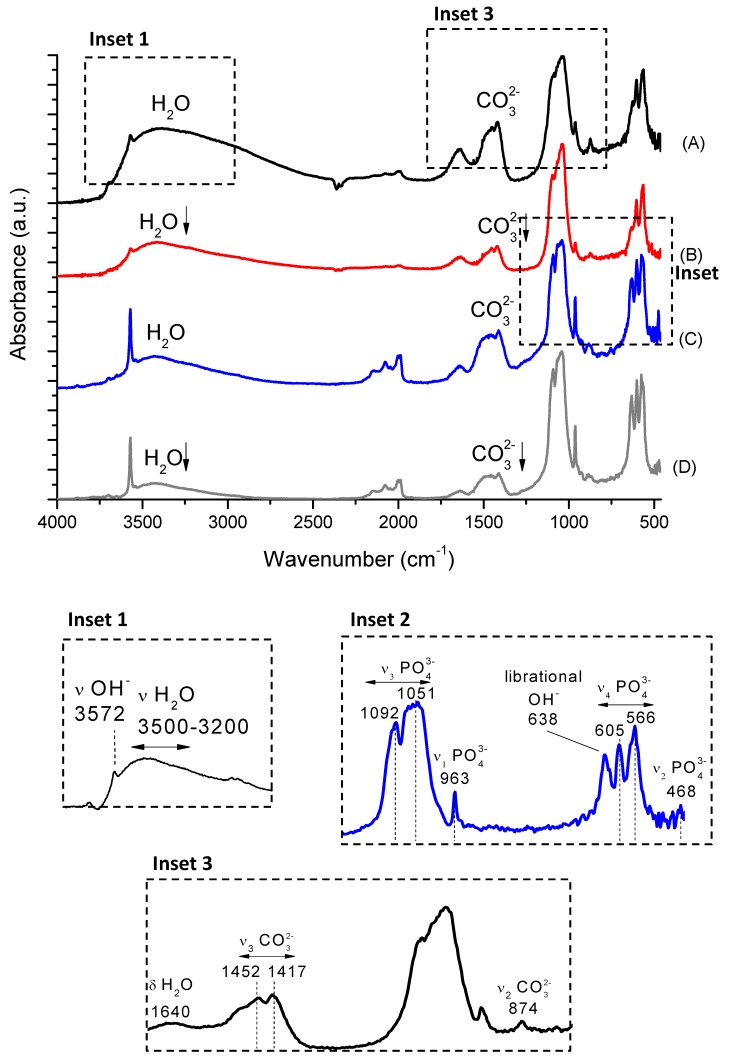
FTIR spectra of HA_110 (**A**); Nb-HA_110 (**B**); HA_900 (**C**) and Nb-HA_900 (**D**).

FTIR spectra of the synthesized samples are shown in Figure 7 (HA_110 (A), Nb-HA_110 (B), HA_900 (C), and Nb-HA_900 (D)). They presented the bands of adsorbed water, hydroxyl, phosphate, and carbonate species from characteristic functional groups of HA. The bands at 3572 cm^–1^ (–OH stretching mode) and 638 cm^–1^ (vibrational mode) are assigned to hydroxyl in HA structure. The broad band in the range of 3500–3200 cm^–1^ (stretching) and the band at approximately 1640 cm^–1^ (bending) are associated with physically and chemically adsorbed water that are typical of hydroxyapatites obtained by wet precipitation routes [28]. HA_110 (Figure 7A) and Nb-HA_110 (Figure 7B) samples presented a larger amount of adsorbed molecules of water and these bands are reduced or even disappear upon thermal treatment (HA_900 and Nb-HA_900, Figure 7C,D, respectively) [26,27], in agreement with the thermal analysis results in the previous section. In addition, the relative intensities of the bands associated with adsorbed water decreased in the doped samples indicating the formation of a more pure HA.

The samples showed the typical phosphate ν_3_ PO_4_^3−^ asymmetric mode at 1092 cm^−1^ and 1051 cm^−1^, ν_1_ PO_4_^3−^ band at 963 cm^−1^, ν_4_ PO_4_^3−^ bands at 605 cm^–1^ and 566 cm^−1^ and ν_2_ PO_4_^3−^ mode at 468 cm^–1^ [3]. The bands of PO_4_^3−^ at 605 cm^–1^, 566 cm^–1^, and 468 cm^–1^ also supported the formation of hydroxyapatite [32]. The FTIR spectra of the Nb-doped HA (Figure 7B,D) did not show significant difference between the intensities and wavenumbers of the phosphates bands compared to undoped HA spectra (Figure 7A,C).

The FTIR spectra of HA also revealed typical bands of carbonate groups (CO_3_^2−^), indicating the formation of carbonated hydroxyapatite. The living bone tissues have partially carbonated calcium phosphate phases and the presence of carbonates in calcium phosphate samples has been extensively reported in the literature mainly associated with carbon dioxide gas from air incorporated *via* aqueous reaction route during the synthesis [33]. There are two different CO_3_^2−^ incorporation lattice sites into HA identified as type-A (carbonate ions at OH^–^ sites) or type-B (carbonate ions at PO_4_^3−^ sites) [34]. Type-A carbonated HA shows the bands of CO_3_^2−^ at 1540–1560 cm^−1^ (ν_4_) and type-B presents bands at 1415–1460 cm^−1^ (ν_3_) and 875 cm^–1^ (ν_2_). The vibrations at approximately 1546 cm^–1^ and 1464 cm^–1^ can be used as IR signature band of type-A and type-B substitution, respectively [35,36]. The HA_110 and Nb-HA_110 (Figure 7A,B, respectively) presented CO_3_^2−^ ions bands at 1452 cm^−1^, 1417 cm^−1^ and 874 cm^–1^ showing the anionic substitution from phosphate groups by carbonates (B-type HA). In the sintered samples (HA_900 and Nb-HA_900, Figure 7C,D) the ν_3_ CO_3_^2−^ band was observed (1415–1460 cm^−1^) and the carbonate band at 875 cm^–1^ almost disappeared indicating the presence of carbonates mainly at the surface of the bioceramic [28]. As a consequence, a more pure HA was obtained after calcination of samples at 900 °C. The carbonate that was incorporated during the syntheses at room temperature was partially removed by heating as indicated by the results of thermal analysis and by the decrease/disappearance of the bands in FTIR spectrum. Additionally, the comparison between Nb-doped and undoped HA indicated a relative decrease of intensity of carbonate bands in Nb-modified samples. This could be a consequence of the substitution of phosphate groups by negatively charged niobate species reducing the available sites for further CO_3_^2−^ incorporation into the lattice. Yet, the vibrational contributions of niobium oxides (oxyanions) or hydroxides were not observed, most likely due to their weak signal at low concentrations and overlapping with other groups out of the detectable range in the FTIR spectra [37,38].

### 2.5. Morphological Analysis

**Figure 8:**
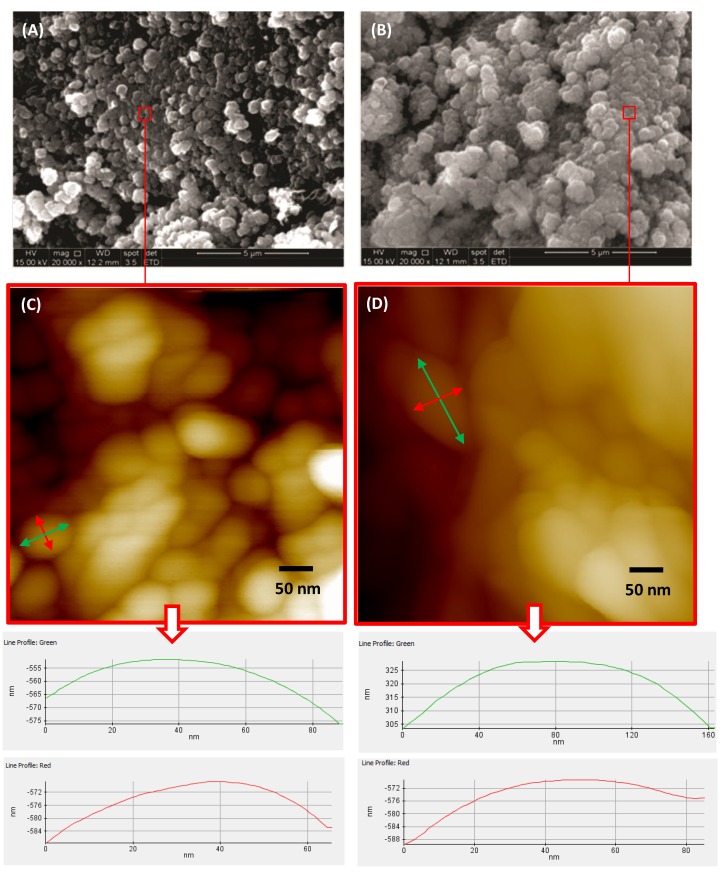
Typical SEM images of HA_110 (**A**) and HA_900 (**B**); AFM images of HA_110 (**C**) and HA_900 (**D**); arrows: AFM images with line profiles measuring the size of nanoparticles (bottom).

**Figure 9:**
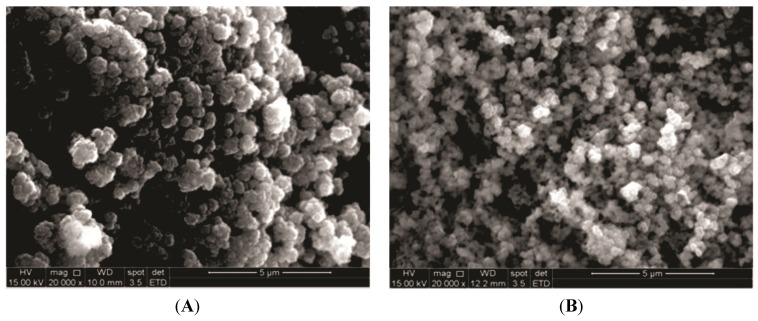
Typical SEM images of bioceramic powders: Nb-HA_110 (**A**) and Nb-HA_900 (**B**).

SEM analysis of the calcium phosphates (Figure 8A,B and Figure 9A,B) showed typical apatite morphology. The images showed the synthesized powders as aggregates, rough, granular to dense [8]. Powders thermally treated at 110 °C (Figure 8A and Figure 9A) revealed the presence of loosely packed particles. After sintering, the HA_900 powder presented more condensed agglomerates (Figure 8B).

AFM images of HA_110 and HA_900 showed the morphological features of these samples at the nanometer scale. The size of the particles and the effect of thermal treatment were accessed by the AFM images. As synthesized HA_110 (Figure 8C) particles showed an apparent spheroidal shape morphology with sizes of approximately 50 nm to 80 nm. Upon heating (HA_900, Figure 8D), the nanoparticles were anisotropic with ellipsoidal-like geometry with sizes of 160 nm length and 80 nm width, which it attributed to particle coalescence.

**Figure 10:**
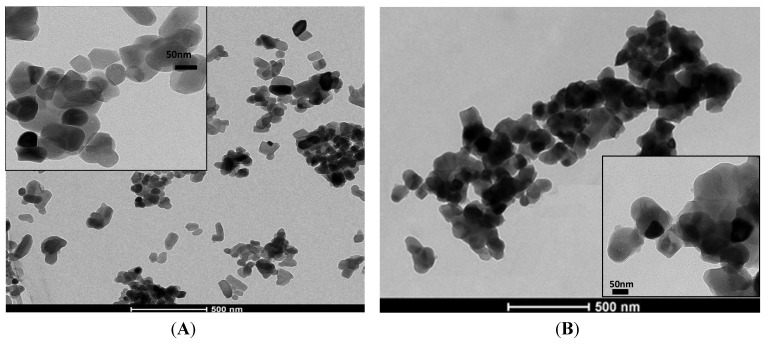
Typical TEM images of HA_900 (**A**) and Nb-HA_900 (**B**).

SEM images of Nb-doped ceramic after sintering (Figure 9B) revealed that the agglomerates of Nb-HA_900 have smaller particle size (180 ± 70 nm) than HA_900 (470 ± 40 nm) and also a denser but more open structure indicating that the incorporation of Nb has changed the morphology of the sintered powder. TEM images have also endorsed these features and revealed the actual size/morphology of the sintered powders. For HA_900 ceramic sample (Figure 10A), the nanoparticles are predominantly agglomerated with some isolated particles that show spheroidal and elongated shapes and sizes typically from 50 to 100 nm (inset in Figure 10A). Nb-doped HA sample (Figure 10B) presented individual particles with spherical shape morphology and size from 60 to 70 nm grouped into micrometric scale agglomerates with a higher degree of coalescence and densification than HA_900. It should be noted that the dimension of the crystals observed by TEM (50–100 nm) was higher than the crystallite sizes calculated from XRD patterns (~36.5 nm, Table 1). That could be attributed to the fact that a given particle observed by TEM would be the result of the aggregation of several crystallites, which could have been differentiated by the XRD analysis [39].

### 2.6. Brunauer–Emmett–Teller (BET) Method

Multipoint BET nitrogen adsorption measurements were performed and the surface area (SA) values of HA_900 and Nb-HA_900 samples were 14.6 ± 0.1 m^2^/g and 8.0 ± 1.2 m^2^/g, respectively. These results validated that Nb-doping caused the densification of HA (~45%). It should be highlighted that SEM and BET are not equivalent techniques as far as porosity analysis is concerned. BET analysis provides precise specific surface area by nitrogen multilayer adsorption measurements yielding important information in studying porosity at the nano-scale. On the other hand, SEM images offer a general qualitative evaluation mostly from the surface, which is dependent on the magnification and cannot access nano-size porosity due to limitation in resolution of the technique.

### 2.7. In Vitro Cytocompatibility Assays

**Figure 11:**
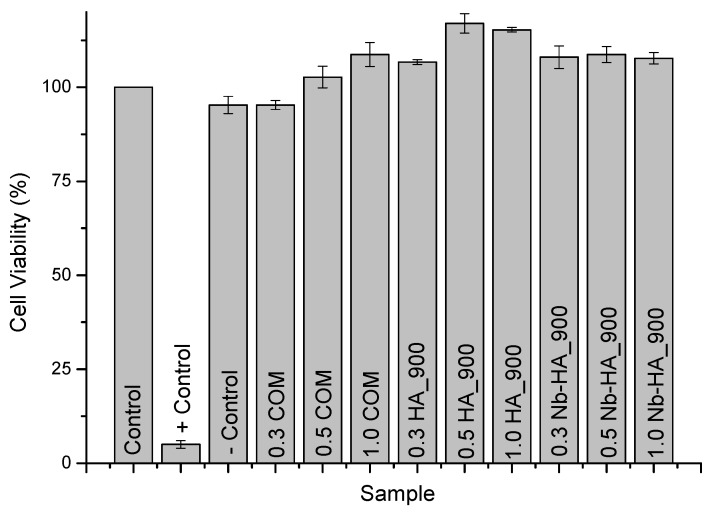
Results of cell viability assay by MTT after 72 h.

Mitochondrial activity of human osteogenic cells (SAOS) cells was assessed by 3-(4,5-dimethylthiazol-2yl) 2,5-diphenyl tetrazolium bromide (MTT) and resazurin analysis. These assays are specifically used and widely accepted for evaluating the toxicity of the bioceramic materials by the analysis of cell viability [40,41]. The results of MTT assay after 72 h of incubation with the cells with HA and Nb-doped HA are presented in Figure 11. It can be observed in the cell viability responses no difference or even a statistically significant increase in cell proliferation when compared to the control condition (reference material).

**Figure 12:**
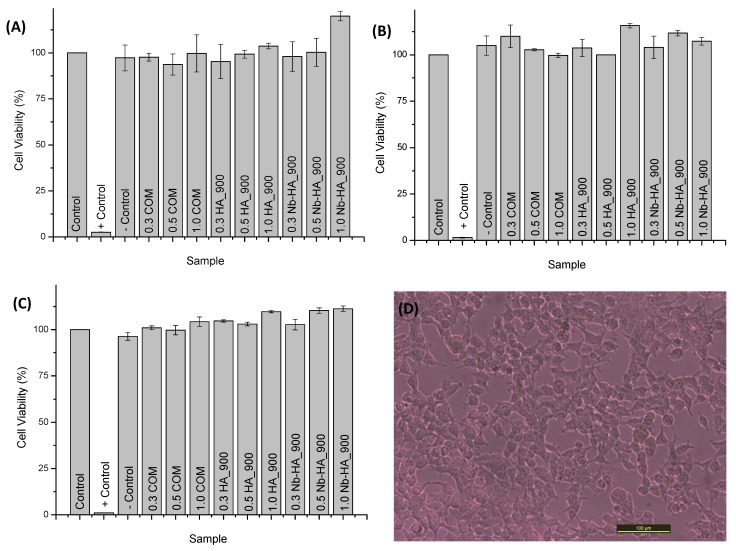
Results of cell viability assay by Resazurin after 24 h (**A**); 48 h (**B**); and 72 h (**C**); SAOS cell viability results after exposure to Nb-HA_900 (**D**).

Additionally, similar results were found in the resazurin assay with no toxicity verified towards synthesized ceramics and commercial product (Figure 12). The comparison of cell viability of CaP ceramics with and without niobium indicates similar behavior. As cell attachment and viability are influenced by surface area [42], this comparable biocompatibility result indicates that Nb-doped ceramics may present a biological response of bone cells better than unmodified HA once its surface area is almost two times smaller as measured by BET assay. Thus, these results have given strong supporting evidence about the ability of the HA_900 and Nb-HA_900 to support proliferation of cells with potential to be tested *in vivo* as a bioceramic alternative for future applications in bone replacements and repair.

## 3. Experimental Section

### 3.1. Materials

All of the reagents and precursors, calcium hydroxide (Ca(OH)_2_, Riedel de Haen, Seelze, Germany), calcium phosphate monobasic monohydrate (Ca(H_2_PO_4_)_2_·H_2_O, Synth, Diadema, Brazil), and niobium chloride (NbCl_5_, Aldrich, St. Louis, MO, USA) were used as-received. Deionized water (DI water, Millipore Simplicity™, Darmstadt, Germany) with resistivity of 18 MΩ·cm was used in the preparation of all solutions. All of the preparations and synthesis were performed at room temperature (23 ± 2 °C) unless specified.

### 3.2. Synthesis of Calcium Phosphate

CaP with 10.0 mol% Nb and without niobium were synthesized from aqueous precipitation route and entirely conducted at room temperature, similar to the procedure reported by our group [43]. The simplified chemical reaction is represented in Equation (1).

7 Ca(OH)_2(susp)_ + 3 Ca(H_2_PO_4_)_2_·H_2_O_(susp)_ → Ca_10_(PO_4_)_6_(OH)_2_ ↓ + 15 H_2_O (1)

A 0.3 mol L^–1^ Ca(OH)_2_ and 0.12 mol L^–1^ calcium phosphate monobasic monohydrate Ca(H_2_PO_4_)_2_·H_2_O suspension was prepared and vigorously stirred for 10 min. The Ca(H_2_PO_4_)_2_·H_2_O suspension was added slowly to the Ca(OH)_2_ suspension and the mixture was magnetically stirred for 1 h. In the sequence, this mixture was aged for 24 h at room temperature. The supernatant was decanted from the solid material. The precipitate was subjected to vacuum filtering using filter paper (25 μm) adapted in a Büchner funnel, washed 3 times with deionized water and filtered again. For niobium modified bioceramics, NbCl_5_ ethanolic solution was added to Ca(H_2_PO_4_)_2_ H_2_O suspension before mixing with calcium hydroxide suspension replacing the amount of 1.0 mol% calcium divalent cation and the same steps of the process were followed. Then, the precipitates were dried at 110 °C for 24 h (HA_110 and Nb-HA_110 samples) and sintered at 900 °C for 3 h (HA_900 and Nb-HA_900 samples). The choice of sintering at 900 °C was based on the literature, aiming at producing monophasic HA to be used as the reference material [43] and minimizing dehydration process during the heating process [44].

### 3.3. Characterization of Calcium Phosphates

The synthesized apatites were characterized by scanning electron microscopy (SEM, JSM 35C, JEOL, Tokyo, Japan) coupled with energy dispersion X-ray spectroscopy (EDX, Voyager EDS 3050, NORAN, Tokyo, Japan) for morphological characterization of samples and element chemical analysis. Before examination, samples were coated with a thin carbon film. Additionally, the concentration of Nb in the precipitate was obtained by wavelength dispersive X-ray fluorescence spectrometry (WD-XRF). This technique can provide high quality determinations of Nb at low level concentrations (*i.e.*, μg g^−1^) comparable with data from more sensitive analytical techniques like ICP-MS (inductively coupled plasma-mass spectrometry) [45]. Calibration curve was prepared using pure NbCl_5_ (Aldrich, St. Louis, MO, USA) as standard and boric acid as diluent. XRF measurements were performed on a PW 2400 spectrometer (PHILIPS, Almelo, The Netherlands) and niobium concentrations were determined from the Nb Kα line intensity.

X-ray diffraction (XRD) patterns were recorded using a X′Pert diffractometer (PANalytical, Cambridge, UK, Cu-Kα radiation with λ = 1.5406 Å). Measurements were performed in the 2θ range of 5° to 60° with steps of 0.06° to identify precipitate phases and estimate crystallinity. The average crystallite size (D_hkl_) of CaP precipitates was estimated from the Scherrer’s equation (Equation (2)) [3].

(2) $${\text{D}}_{\text{hkl}}=\frac{\text{kλ}}{\text{βcosθ}}$$

where, D_hkl_ is the average crystallite size (nm); β is the full width of the peak at half of the maximum intensity (2θ); λ is the wavelength of X-ray radiation and k is the shape coefficient (value between 0.9 and 1).

Lattice parameters a and c were calculated from peaks (300) and (002), respectively, using the standard hexagonal unit cell plane spacing relationship (Equation (3)) [4].

(3) $$\frac{1}{{\text{d}}^{2}}=\frac{4}{3}\left(\frac{{\text{h}}^{2}+\text{hk}+{\text{k}}^{2}}{{\text{a}}^{2}}\right)+\frac{{\text{l}}^{2}}{{\text{c}}^{2}}$$

where, d is the distance between adjacent planes in the set of Miller indices (hkl). 

The nanostructural morphological analysis of the ceramics was conducted based on images obtained *via* transmission electron microscopy (TEM) using a Tecnai G2-20-FEI microscope (FEI Company, Hillsboro, OR, USA) at an accelerating voltage of 200 kV. EDX spectra were collected for element chemical analysis. The TEM samples were prepared by dropping an aliquot of ethanolic dispersion of ceramic samples (1.0 g L^–1^ magnetically stirred for 5 h) onto a holey carbon grid before the analysis. In addition, atomic force microscopy (AFM) was conducted with an XE-70 instrument (Park System, Suwon, Korea) operating in non-contact mode. The scanning rate was 1.0 Hz, and the images were acquired with a 512 × 512 pixel resolution. The samples were prepared by dropping the CaP ethanolic dispersion onto a mica muscovite substrate. The areas were randomly selected for statistical purposes.

Thermogravimetric (TG) and differential scanning calorimetry (DSC) analyses were performed using SDT Q-600 simultaneous TGA/DSC instrument (TA Instruments Co., New Castle, DE, USA). Samples of approximately 8.5 ± 1.0 mg were used for the experiments at a heating rate of 20 °C min^–1^ (range from 20 to 1000 °C). The samples were loaded into an open alumina crucible. TG and DSC curves were recorded simultaneously with 0.1 μg sensitivity. The thermal analyses were performed under the continuous flow of dry nitrogen gas (100 mL min^−1^).

CaP and niobium-doped CaP were analyzed by diffuse reflectance infrared Fourier transform spectroscopy (DRIFTS) method (Nicolet 6700, Thermo Fischer, Waltham, MA, USA) over the range of 400 to 4000 cm^−1^ using 64 scans and a 2 cm^–1^ resolution with the subtraction of KBr background. The samples were mixed in a ratio of 1% (wt%) to KBr powder dried at 110 ± 5 °C. for 2 h.

Surface area of alumina powders was characterized by multipoint BET (Brunauer, Emmett, and Teller) nitrogen adsorption method (NOVA^®^-1200 V.5.25, Quantachrome Instruments, Boynton Beach, FL, USA), with minimum 8 h of degassing at 110 ± 5 °C.

### 3.4. In Vitro Characterization Assays by MTT and Resazurin

Human osteogenic cells (SAOS) were kindly provided by Prof. A. Goes (Department of Immunology and Biochemistry, Federal University of Minas Gerais, Brazil). The cells were cultured in Dulbecco’s modified eagle medium (DMEM) with 10% FBS (fetal bovine serum), penicillin G sodium (10 units mL^–1^), streptomycin sulfate (10 mg mL^–1^) and 0.25 anfotericin-b all from Gibco BRL (New York, NY, USA) in a humidified atmosphere of 5% CO_2_ at 37 °C. Cells used for the experiments were in passage 6.

Biological tests were conducted according to ISO standards 10993-5:1999 (Biological evaluation of medical devices; Part 5: tests for *in vitro* cytotoxicity). SAOS cells were plated (1 × 10^4^ cells/well) in 24-well plates. Cell populations were synchronized in serum free medium for 24 h, after this period, the medium was aspirated and replaced with medium containing FBS. Samples were prepared using different concentrations of ceramics (0.3 mg mL^–1^, 0.5 mg mL^–1^, and 1.0 mg mL^–1^) and they were sterilized with UV radiation for 40 min. Controls had been used with cells and DMEM medium with 10% of FBS, positive control PBS (phosphate buffer saline) 10×, and as negative control chips sterile polypropylene Eppendorf (1 mg mL^−1^, Eppendorf, Hamburg, Germany). Commercially purified HA (HAP-91^®^, JHS–Laboratório Químico, Belo Horizonte, Brazil) was used as reference material. Results were presented as means ± standard deviation. Statistical analysis was performed using one way analysis of variance (ANOVA) using the Bonferroni method (n = 3, GraphPadPrism, GraphPad Software, Inc., La Jolla, CA, USA).

For the MTT assay, after 72 h, all of the media were aspirated and replaced with 210 µL culture medium with serum to each well. Then, 170 µL of MTT (5 mg·mL^–1^, Sigma-Aldrich, St. Louis, MO, USA) were added to each well and incubated for 4 h in an oven at 37 °C and 5% CO_2_ followed by addition of 100 µL of isopropanol containing 4% HCl. In the sequence, 100 µL were removed from each well and transferred to a 96-well plane and the absorbance (Abs) was recorded at 595 nm filter (iMark™ microplate absorbance reader, Bio-Rad, Hercules, CA, USA). The measured values were expressed as percentage of viable cells according to Equation (4).

(4) $$\text{Cell viability}=\;\frac{\text{Abs of sample and cells}}{\text{Abs of control}}\times 100\text{\%}$$

In the resazurin assay, after 24, 48, and 72 h, all of the media were aspirated and replaced by 900 µL of culture medium with serum in each well. Then, 100 µL of resazurin stock solution (0.1 mg mL^–1^, Sigma-Aldrich, St. Louis, MO, USA) were added to each well and incubated for 12 h in an oven at 37 °C and 5% CO_2_. In the sequence, 100 µL were removed from each well and transferred to a 96-well plate and the absorbance values were recorded at the wavelengths of 570 nm and 595 nm (iMark™ microplate absorbance reader, Bio-Rad, Hercules, CA, USA). The measured values were expressed as percentage of viable cells according to Equation (4).

## 4. Conclusions

In summary, the results demonstrated that Nb(V) anionic species (e.g., niobates) were effectively incorporated in the doped hydroxyapatite by partial substitution of phosphate groups, leading to the modification of the original crystalline structure. In addition, it caused a refinement in the average particle size and a higher densification of the bioceramic after sintering compared with the undoped HA. Regarding the cytocompatibility *in vitro*, no toxicity was verified on MTT and resazurin assays for the Nb-alloyed HA with cell viability response similar to that of pure HA reference biomaterial. Thus, it can be affirmed that a novel Nb-doped HA bioceramic was successfully produced at room temperature using aqueous co-precipitation process, which offers promising biomaterial as potential bone replacement and repair in the future.
